# Sexual behavior of HIV-positive adults not accessing HIV treatment in Mombasa, Kenya: Defining their prevention needs

**DOI:** 10.1186/1742-6405-9-9

**Published:** 2012-03-19

**Authors:** Avina Sarna, Stanley Luchters, Melissa Pickett, Matthew Chersich, Jerry Okal, Scott Geibel, Nzioki Kingola, Marleen Temmerman

**Affiliations:** 1 Population Council, 142 Golf Links, New Delhi 110048, India; 2 International Centre for Reproductive Health, Department of Obstetrics and Gynaecology, Ghent University, Ghent, Belgium; 3 UCLA Center for Health Policy Research, Los Angeles, USA; 4 Centre for Health Policy, School of Public Health, Faculty of Health Sciences, University of the Witwatersrand, Johannesburg, South Africa; 5 Population Council, Nairobi, Kenya; 6 International Centre of Reproductive Health, Mombasa, Kenya

**Keywords:** PLHIV, Prevention of sexual transmission of HIV, Sexual behavior, Unsafe sex, Africa

## Abstract

**Background:**

HIV spread continues at high rates from infected persons to their sexual partners. In 2009, an estimated 2.6 million new infections occurred globally. People living with HIV (PLHIV) receiving treatment are in contact with health workers and therefore exposed to prevention messages. By contrast, PLHIV not receiving ART often fall outside the ambit of prevention programs. There is little information on their sexual risk behaviors. This study in Mombasa Kenya therefore explored sexual behaviors of PLHIV not receiving any HIV treatment.

**Results:**

Using modified targeted snowball sampling, 698 PLHIV were recruited through community health workers and HIV-positive peer counsellors. Of the 59.2% sexually-active PLHIV, 24.5% reported multiple sexual partners. Of all sexual partners, 10.2% were HIV negative, while 74.5% were of unknown HIV status. Overall, unprotected sex occurred in 52% of sexual partnerships; notably with 32% of HIV-negative partners and 54% of partners of unknown HIV status in the last 6 months. Multivariate analysis, controlling for intra-client clustering, showed non-disclosure of HIV status (AOR: 2.38, 95%CI: 1.47-3.84, p < 0.001); experiencing moderate levels of perceived stigma (AOR: 2.94, 95%CI: 1.50-5.75, p = 0.002); and believing condoms reduce sexual pleasure (AOR: 2.81, 95%CI: 1.60-4.91, p < 0.001) were independently associated with unsafe sex. Unsafe sex was also higher in those using contraceptive methods other than condoms (AOR: 5.47, 95%CI: 2.57-11.65, p < 0.001); or no method (AOR: 3.99, 95%CI: 2.06-7.75, p < 0.001), compared to condom users.

**Conclusions:**

High-risk sexual behaviors are common among PLHIV not accessing treatment services, raising the risk of HIV transmission to discordant partners. This population can be identified and reached in the community. Prevention programs need to urgently bring this population into the ambit of prevention and care services. Moreover, beginning HIV treatment earlier might assist in bringing this group into contact with providers and HIV prevention services, and in reducing risk behaviors.

## Background

HIV transmission remains a significant global concern; in 2009 there were an estimated 2.6 million new infections globally [1]. At the end of 2009, about 36% of the 15 million people in need of antiretroviral treatment (ART) in low- and middle income countries were receiving ART [1].

People living with HIV (PLHIV) who receive ART are in regular contact with health workers and presumably exposed to prevention messages and commodities. Indeed, several studies have documented a reduction in sexual risk behaviors among PLHIV after initiating ART [2-6]. At the same time, studies have shown that PLHIV accessing HIV care services, but not receiving ART, have higher sexual risk behaviors and unprotected sex than those taking ART, even though both groups have contact with health workers and exposure to prevention messages [7-10]. A major gap, however, is evidence about the patterns of sexual behavior among PLHIV in the community who are not receiving ART and are either accessing HIV care services infrequently or not at all. Although newly diagnosed HIV-positive persons are advised to visit treatment centres for routine follow-up, many PLHIV choose not to. HIV related stigma, denial and disclosure concerns constitute important barriers to accessing care [11,12]. The only contact with health services for these people might well be post-test counselling at the time of testing HIV-positive. At the same time, PLHIV are also exposed to HIV prevention messages through mass media and community awareness programs that presumably also influence their knowledge and behaviors.

Studies of the determinants of unprotected sex in HIV-infected people suggest that a range of factors can operate individually or interact to influence sexual behavior [13]. Intention and self-efficacy regarding safe sex; [14,15] myths around condom use; dilemmas around disclosure of HIV status to partner(s) and fears of subsequent rejection; [14,16-20] and motivation to protect partners as well as themselves against re-infection with a new HIV strain or another sexually transmitted infection play an important role in effecting safe sex [13,20]. Partner attitudes and willingness to use condoms, complicated by partner status and willingness to be tested for HIV add further dimensions to safe sex practices [4,18,19,21]. Furthermore, a desire for children may lead to PLHIV ignoring the risks of unprotected sex [19,22,23].

In Kenya, in 2009, an estimated 1.3 to 1.6 million persons were living with HIV and an estimated 40% of PLHIV with advanced disease who are eligible for treatment were not receiving ART [1,24]. At the same time a large number of PLHIV do not yet require ART. Many of these PLHIV are likely to be outside the ambit of regular health care and prevention services. An estimated 100,000 new HIV infections occurred in 2009 in Kenya, highlighting the need for prevention efforts to focus on sexual risk behaviors of PLHIV, including those not accessing HIV care services. In this paper we examine the sexual risk behaviors of PLHIV in the community who were not receiving ART or co-trimoxazole prophylaxis.

## Methods

Study participants were recruited for a cross-sectional survey, using modified targeted snowball sampling that uses outreach workers to recruit participants from identified geographic areas and populations of interest [25,26]. In classical snowball sampling a small number of individuals (typically between 4-6 persons initially) from a particular group of interest are identified, who then serve as 'seeds' to identify and recruit peers, that is, individuals who engage in the same type of risk behaviors, to be included into the study sample [27]. These, initial 'seeds' are often selected by program or study staff via convenience sampling. 'Seeds' can recruit an unlimited number of peers from their network till the desired sample size is achieved or sample saturation takes place. A drawback of this method is that the sample obtained is influenced by characteristics of the initial seeds, the size of their personal network and their ability to reach more cooperative subjects, with a possibility of sampling bias [27]. By contrast, modified targeted sampling, aims to overcome some of the limitations of snowball sampling by including an initial ethnographic assessment aimed at identifying the various networks or subgroups that might exist in a given setting [25]. Participants are then recruited through the active efforts of street outreach workers, using snow-ball sampling. CHWs and PTC counsellors are familiar with the community they serve, the socio-demographic profile of the community and clients, and can help reach PLHIV; we used this cadre of health workers to recruit our study sample. Health workers identified PLHIV in their community and asked these PLHIV to bring in others they knew. As our previous study showed us that PLHIV in Mombasa are relatively isolated due to stigma and disclosure concerns [28], health workers were permitted to add new 'seeds' if PLHIV were unable to bring in peers.

Participants were recruited by community health workers (CHWs) and HIV-positive peer counselors (PCs) from post-test clubs. To reduce biases related to the recruiter and the initial sample, especially over representation of more cooperative subjects and respondents with larger networks, the number of clients each health worker could bring into the study was restricted. Four CHWs from each of Mombasa's four districts (n = 16) were each asked to recruit 20 PLHIV; and five PCs from each of eight post-test centers (n = 40) across the four districts were each tasked with recruiting 12 PLHIV. HIV-positive adults who were 18 years or older, not currently taking ART or co-trimoxazole prophylaxis were eligible to participate.

Recruitment followed a detailed protocol on approaching PLHIV, maintaining confidentiality and verifying the participant's HIV-positive status by checking the referral card issued by a VCT center, or HIV clinic registration card or HIV/CD4 cell test results. Each participant received Ksh 200 (1USD = +/-75Ksh) as compensation for their time and transport. CHWs and PCs received Ksh 100 per participant recruited to cover their transport costs. Ethical approval was obtained from the Kenyatta National Hospital's Ethics Committee and Institutional Review Board of the Population Council. Written informed consent was obtained from all participants. After data collection was completed, project staff worked with CHWs and PTC counsellors to counsel each of the clients they had recruited to return to the HIV clinic for further follow up, care and ART.

Data were collected using structured questionnaires administered in Swahili by trained research assistants. Demographic variables were categorized and time since HIV diagnosis was classified as less than 12 months, 12-24 months and > 24 months. Contraception was categorized as: male/female condoms for contraception, other family planning (FP) methods (intra-uterine device, hormonal methods, permanent methods, diaphragm, foam/jelly, or rhythm) and no contraception. Disclosure of HIV status to a sexual partner was recorded as a binary yes/no variable. Perceived stigma was assessed using an adapted Berger's Stigma Scale (Cronbach's alpha of adapted scale: 0.81) and was categorized as minimal or low (16- 40), moderate (41-52) or high stigma (53-64) [29,30]. The recall reference period for sexual behavior was the previous 6 months, with data collected on: having had sexual intercourse, number of sexual partners, type of partners, partner's HIV status and disclosure of own status to partners. A regular partner was defined as a spouse or cohabiting partner, or a long-term friend with whom the respondent has sex frequently. A partner with whom the respondent was not living and had sex once or rarely was classified as a casual partner. Commercial or transactional partners were those where money or gifts were exchanged for sex.

To assess transmission concerns, participants were asked a binary question: "Are you worried about transmitting HIV to this partner?" Attitudes to condom use were assessed with six statements: "I am tired of always having to make sure that I use a condom every time I have sex","using condom reduces physical pleasure from sex","if a cure were discovered I would stop using condom", "using a condom takes away the romance from sex", "condoms are effective in preventing HIV and STDs" and "condom should only be used to prevent pregnancy and not HIV"- responses were scored on a 4-point Likert scale, ranging from strongly agree, agree, disagree, to strongly disagree. Respondents were also given the option of saying don't know. These statements were adapted from other studies [13,31,32] and pretested, validated and used in a previous study with PLHIV in Mombasa [2,19]. STI events were self-reported episodes of genital discharge or genital ulcer in the last 6 months (laboratory confirmation was unavailable). Participants were asked about the number of biological children they had. Fertility intentions were assessed by asking participants about the intention to have children in the future. Unprotected sex (UPS) was defined as inconsistent condom use with any partner in the past 6 months. Unsafe sex (US), a subset of UPS and the primary outcome for multivariate analysis to determine predictors, was defined as inconsistent condom use with HIV-negative or unknown status partners in the last 6 months. UPS at last sex and US at last sex were also reported.

### Data management and statistical analysis

Data were entered into handheld computers (Dell Axim × 51) and then uploaded into Microsoft Access 2003 using Perseus 7.0.044 software. The data were analyzed on two levels (respondent-level and partner-level) using Intercooled Stata 8.0 (Stata Corporation, College Station, Texas, USA).

Respondent-level analysis compared demographic and behavioral characteristics of male and female participants. Unpaired Student's *t *test and the Mann-Whitney U test compared continuous variables with normal or non-normal distributions respectively, and a chi-square test identified differences between categorical variables. Unadjusted Mantel Hanzel odds ratios were reported.

Analysis at the level of sexual partner included data for up to 6 partners for each respondent in the last 6 months. Univariate logistic regression analyses were performed to identify associations between variables of interest and US at 6 months and last sex. Variables significant, at alpha level of 0.05, on univariate regression were included in the multivariate model [33]. Although sex of the respondent was not associated with unsafe sex in univariate analysis, it was forced into the model as socio-demographic characteristics varied markedly between women and men (Table 1). Also, a priori, disclosure of HIV status and type of partner were included in initial models, based on previous evidence of association with unprotected sex [7,34-37]. As a participant's sexual behavior with one partner may not be independent from her or his behavior with other partners, we controlled for multiple observations on sexual partners reported by the same study participant (intra-client clustering). Multiple partners of the same participant were also included as separate units of analysis. We adjusted the standard errors for clustering on the participant's ID in both univariate and multivariate logistic regression analyses. A main effects model was used to fit the multivariate model [38]. Separate multivariate models were developed for US at 6 months and at last sex.

**Table 1:** Participant characteristics: HIV-positive adults not receiving ART, Mombasa, 2007

Variable	Total (n = 698)	Males (n = 164)	Females (n = 534)	P^a^
**Age: **median (IQR)	33.5 (28-39)	34.5 (29-42)	33 (28-38)	0.02^b^
**Highest education level:**% (n)				
No education	7.3 (51)	3.7 (6)	8.4 (45)	0.04
Primary	59.2 (413)	54.9 (90)	60.5 (323)	
Secondary	31.1 (217)	38.4 (63)	28.8 (154)	
University	2.4 (17)	3.1 (5)	2.3 (12)	
**Marital status:**% (n)				
Married or cohabiting	34.4 (240)	40.9 (67)	32.4 (173)	< 0.001
Never married	21.1 (147)	32.9 (54)	17.4 (93)	
Divorced or separated	20.4 (143)	15.8 (26)	21.9 (117)	
Widowed	24.1 (168)	10.3 (17)	28.2 (151)	
**Employment status:**% (n)				
Employed	75.9 (530)	82.9 (136)	73.8 (394)	0.02
**Type of HIV testing facility used:**% (n)				
Government health facility	80.7 (563)	77.4 (127)	81.7 (436)	< 0.001
Private medical centre	15.3 (107)	12.2 (20)	16.3 (87)	
Other	4.0 (28)	10.4 (17)	2.1 (11)	
**Time since diagnosis:**% (n)^c^				
0-11 months	43.1 (301)	50.0 (82)	41.0 (219)	< 0.001
12-23 months	19.5 (136)	22.6 (37)	18.5 (99)	
24+ months	33.4 (233)	23.2 (38)	36.5 (195)	
**Attends HIV clinic:**% (n)				
Yes	23.4 (163)	16.5 (27)	25.5 (136)	0.02
No	76.7 (535)	83.5 (137)	74.5 (398)	
**Perceived level of stigma:% **(n)				
Low	16.2 (113)	18.9 (31)	15.4 (82)	0.5
Moderate	68.8 (480)	67.7 (111)	69.1 (369)	
High	15.0 (105)	13.4 (22)	15.5 (83)	
**Drink alcohol weekly:**% (n)				
Yes	26.9 (188)	34.2 (56)	24.7 (132)	0.02
**Has ever used drugs:**% (n)				
Yes	31.5 (220)	68.5 (104)	21.7 (116)	< 0.001
**Have biological children:**% (n)				
Yes	81.7 (570)	65.9 (108)	86.5 (462)	< 0.001
**Want to have children:**% (n)				
No	74.8 (522)	61.6 (101)	78.8 (421)	< 0.001
**Using family planning method:**% (n)^d^				
No	54.8 (280)	45.5 (49)	56.2 (237)	0.072
**Reported correct knowledge:**%(n)				
HIV cannot spread through mosquitoes	72.8 (508)	72.6 (119)	72.8 (389)	0.93
HIV cannot spread through shared utensils	84.7 (591)	83.5 (137)	85.0 (454)	0.62
HIV can be transmitted from a mother to child	91.5 (639)	90.2 (148)	91.9 (491)	0.70
Treatment can reduce mother-to-child transmission	61.1 (425)	55.5 (91)	62.8 (334)	0.20
HIV+ person can be re-infected with a new virus	68.6 (479)	64.6 (106)	69.9 (373)	0.45

^a ^*X*^2 ^test unless indicated

^b ^Mann-Whitney *U *test

^c ^n = 671; 28 respondents did not provide information on the time since diagnosis

^d ^of those not wanting children/more children

ART: antiretroviral therapy; IQR: interquartile range

## Results

Between May and August 2007, 748 PLHIV were identified by CHWs and PCs; 28 persons were found ineligible (receiving treatment) and 720 PLHIV were interviewed. Data from 22 participants were lost due to technical failures with the hand-held computers, leaving data on 698 participants for analysis.

Median age of participants was 33.5 years (IQR = 28-33). Twenty-three percent (163/698) of participants reported visiting HIV clinics (34.4% visited monthly, 16.6% every two to six months, 20.2% when sick and 28.8% off and on). Differences were observed in socio-demographic characteristics between female and male respondents [Table 1]. Women were more likely than men to be widowed (OR 3.40; 95%CI: 1.98-5.88; p < 0.001); to attend HIV clinic (OR 1.73; 95%CI: 1.10-2.74; p = 0.017) and be unemployed (OR 1.73; 95%CI: 1.10- 2.71; p = 0.018). Women were also less likely to drink alcohol each week (OR: 0.63; 95%CI: 0.43-0.93; p = 0.017) or to report ever using drugs (OR: 0.16; 95%CI: 0.11-0.24; p < 0.001). Women knew their HIV-status for longer periods than men. Participants recruited by CHWs and by PCs had a similar age, sex, education and employment status (data not shown).

Participants were asked about their reasons for not taking ART; multiple responses were permitted. About a quarter (27.9%; n = 195) reported high CD4 cell counts that made them ineligible for ART; 16.8% (n = 117) did not want to start ART; 11.2% (n = 78) reported they were afraid of side-effects; 7.7% (n = 54) did not know where to access treatment; 2.9% (n = 20) complained that treatment was expensive; 2.4% (n = 17) were taking herbal remedies and 2.1% (n = 15) had unfavourable beliefs about ART, such as, 'ARVs can make you mad', 'ARVs kill you faster', 'ARV are brought by donors when they stop it will be the end of your life', and ARVs make you sicker'.

### Sexual activity

In the 6 months preceding the survey, 59.2% percent of participants were sexually active; similar in females and males [Table 2].

**Table 2:** Sexual behavior among HIV-positive adults not receiving ART in Mombasa, Kenya 2007

All Respondents				
	**Total (n = 698)**	**Males** (n = 164)	**Females** (n = 534)	**P Value^a^**

**Lifetime no. of partners: **median (IQR)^b^	5 (3,10)	14 (6,25)	4 (3,8)	< 0.001

**Sexually active in past 6 months:**% (n)	59.2 (413)	55.5 (91)	60.3 (322)	0.27

**Sexually Active Respondents**				

	**Total** **(n = 410)^c^**	**Male** **(n = 90)**	**Female** **n = 320)**	

**No. of partners in past 6 months:**% (n)^d^				

One partner	75.5 (308)	54.4 (49)	81.5 (259)	

More than one partner	24.5 (100)	45.6 (41)	18.6 (59)	< 0.001

**Sex of partner:**% (n)				

Only male	79.8 (327)	12.2 (11)	98.8 (316)	

Only female	18.8 (77)	84.4 (76)	0.3 (1)	

Both male & female	1.5 (6)	3.3 (3)	0.9 (3)	< 0.001^e^

**Type of partner:**% (n)				

Only regular	76.3 (313)	62.2 (56)	80.3 (257)	

Only casual	8.5 (35)	11.1 (10)	7.8 (25)	

Only sex worker	3.2 (13)	6.7 (6)	2.2 (7)	

Multiple types	12.0 (49)	20.0 (18)	9.7 (31)	0.002

**Sexually Active Respondents (partner level analysis: n = 616)**				

	**Total number of partners reported by 410 sexually active participants**	**Number of partners 90 sexually active men reported**	**Number of partners 320 sexually active women reported**	**P Value^a^**

**Partners of respondents**	n = 616	n = 179	n = 437	

**Sex of partner:**% (n)				

Male	78.1 (481)	26.8 (48)	99.1 (433)	

Female	21.9 (135)	73.2 (131)	0.9 (4)	< 0.001^e^

**Type of partner:**%(n)				

Regular	65.9 (406)	50.8 (91)	72.1 (315)	

Casual	20.8 (128)	23.5 (42)	19.7 (86)	

Sex worker	13.3 (82)	25.7 (46)	8.2 (36)	< 0.001

**Partner HIV status:**% (n)				

Positive	15.3 (94)	17.3 (31)	14.4 (63)	

Negative	10.2 (63)	9.5 (17)	10.5 (46)	

Unknown	74.5 (459)	73.2 (131)	75.1 (328)	0.64

**Disclosure:**% (n)				

Partner knows	37.0 (228)	30.2 (54)	39.8 (174)	

Partner does not know	63.0 (388)	69.8 (125)	60.2 (263)	0.02

^a ^*X*^2 ^test unless indicated

^b ^n = 684; 14 respondents were excluded if they did not know, did not respond, or reported ≥ 800 partners

^c ^n = 410; 3 sexually active respondents did not answer further questions about their sexual partners

^d ^n = 408; 2 respondents did not respond

^e ^Fisher's exact test

ART, antiretroviral therapy; IQR, interquartile range

Males were more likely than female participants to report multiple partners (OR: 3.67; 95%CI 2.18-6.18; p < 0.001) in the last 6 months. Sexually-active male respondents (90/164) reported a total of 179 sexual partners and female respondents (320/534) reported a total of 437 sexual partners over the reference period [Table 2].

While the majority of male (84.4%) and female participants (98.8%) reported heterosexual partners, 15.5% of males (14/90) and 1.2% of females (n = 4/320) reported same sex partners in the last 6 months [Table 2]. Over a quarter of men's sexual partners were males (26.8%; 48/179).

Twenty percent of male participants reported a mix of sexual partners (regular/casual/transactional) compared to 9.7% of female participants (OR: 2.33; 95%CI: 1.23-4.43; p < 0.01). [Table 2] Female participants reported more regular partners than male participants (72.1% vs. 50.8%; OR: 2.50; 95%CI: 1.73-3.61; p < 0.001) while male participants had more casual (23.5% vs. 19.7%; OR: 1.25; 95%CI: 0.82-1.90; p = 0.29) and transactional partners (25.7% vs. 8.2%; OR: 3.85; 95%CI: 2.35-6.30; p = < 0.001) than women (p < 0.001) [Table 2].

Three-quarter of all partners were of unknown HIV status, similar for men and women. Female respondents reported higher disclosure rates to partners than male respondents (39.8% vs. 30.2%.; OR: 1.53; 95%CI: 1.09-2.47; p = 0.02) [Table 2].

### Prevalence of unprotected sex

UPS-6 months (inconsistent condom use with any partner in the last 6 months) was reported in over half (51.9%) the sexual partnerships, more by women than men (55.2% vs. 44.1%; OR: 1.56; 95%CI: 1.09-2.21; p = 0.01) [Table 3]. Males were more likely to report UPS-6 months with their female partners than their male partners (52% vs. 22.9%; OR: 3.63; 95%CI: 1.66-7.95; p = 0.001). Both sexes were more likely to have UPS-6 months with regular partners compared to casual or transactional partners (p < 0.001). Inconsistent condom use in the last 6 months (US-6 months) was reported with 31% of HIV-negative partners (females 30.4% vs. males 35.3%; OR: 0.80; 95%CI: 0.25-2.63; p = 0.72) and with 53.8% of the partners of unknown HIV status (females 57.3% vs. males 45.0%; OR: 1.64; 95%CI: 1.07-2.47; p = 0.02). Patterns of UPS at last sex were similar to those of UPS-6 months (data not shown).

**Table 3:** Prevalence of Unprotected Sex in the past 6 months among sexually-active participants (partner level analysis)

	Total number of partners reported by 410 sexually active respondents n = 616	UPS-6 months Number of partners by 90 sexually active male respondents n = 179	Number of partners reported by 320 sexually active female respondents n = 437
**Total Unprotected Sex:**% (n)	51.9 (320/616)	44.1 (79/179)	55.2 (241/437)
**By sex of partner:**% (n)			
Male	52.4 (252/481)	22.9 (11/48)	55.7 (241/433)
Female	50.4 (68/135)	52.0 (68/131)	0 (0/4)
		p = 0.001	p = 0.04^b^
**By type of partner:**% (n)			
Regular	61.1 (248/406)	59.3 (54/91)	61.6 (194/315)
Casual	34.4 (44/128)	26.2 (11/42)	38.4 (33/86)
Sex worker	34.2 (28/82)	30.4 (14/46)	38.9 (14/36)
	p < 0.001	p < 0.001	p < 0.001
**By partner status:**% (n)			
Positive	56.4 (53/94)	45.2 (14/31)	61.9 (39/63)
Negative	31.8 (20/63)	35.3 (6/17)	30.4 (14/46)
Unknown	53.8 (247/459)	45.0 (59/131)	57.3 (188/328)
	p = 0.003	p = 0.74	p < 0.001
**By disclosure:**% (n)			
Partner knows	53.1 (121/228)	38.9 (21/54)	57.5 (100/174)
Partner does not	51.3 (199/388)	46.4 (58/125)	53.6 (141/263)
know	p = 0.67	p = 0.35	p = 0.43

^a ^*X*^2 ^test unless indicated

^b ^Fisher's exact test

ART, antiretroviral therapy

### Predictors of unsafe sex (inconsistent condom use with HIV negative or unknown status partners)

Risk factors associated with US-6 months were explored (Table 4). In univariate analysis, university level education, more than 12 months since HIV diagnosis, non-disclosure of HIV-status, moderate and high levels of internalized stigma, condom-use fatigue, attending a HIV clinic, knowing that re-infection with a new viral strain is possible, believing that condoms reduce pleasure and using non-condom contraceptive methods were associated with higher risk of US-6 months and were included in the initial model.

**Table 4:** Factors associated with Unsafe Sex in the past 6 months among sexually-active participants, adjusted for intra-client clustering

		US-6 months			
Variable	Prevalence % (n)	Crude Odds (95% CI)	P value	Adjusted Odds (95% CI)	P value
**Sex of respondent**					
Male (n = 179)	36.3 (65)	1.0	---	---	---
Female (n = 437)	46.2 (202)	1.51 (0.88-2.59)	0.14	2.10 (1.13-3.90)	0.018
**Age**					
18-24 years (n = 106)	42.5 (45)	0.85 (0.44-1.65)	0.64		
25-34 years (n = 291)	46.4 (135)	1.0	---		
35-44 years (184)	39.7 (73)	0.76 (0.46-1.26)	0.29		
45+ (n = 35)	40.0 (14)	0.77 (0.31-1.94)	0.58		
**Marital status**					
Married or cohabiting (n = 232)	44.8 (104)	1.0	---		
Never married (n = 184)	41.3 (76)	0.87 (0.49-1.51)	0.61		
Divorced, separated, or widowed (n = 200)	43.5 (87)	0.95 (0.59-1.53)	0.83		
**Highest education level**					
No education (n = 42)	52.4 (22)	1.32 (0.53-3.26)	0.55	0.53 (0.20-1.37)	0.19
Primary (n = 378)	45.5 (172)	1.0	---	---	---
Secondary (n = 179)	39.1 (70)	0.77 (0.47-1.26)	0.30	1.21 (0.63-2.32)	0.56
University (n = 17)	17.7 (3)	0.26 (0.07-0.98)	0.05	0.82 (0.22-3.03)	0.77
**Sex of partner**					
Male (n = 481)	44.3 (213)	1.0	---		
Female (n = 135)	40.0 (54)	0.84 (0.49-1.42)	0.52		
**Type of partner**					
Regular (n = 406)	48.3 (196)	1.80 (0.88-3.68)	0.11		
Casual (n = 128)	33.6 (43)	0.98 (0.41-2.32)	0.96		
Sex worker (n = 82)	34.2 (28)	1.0	---		
**Attends HIV clinic**					
Yes (n = 122)	25.6 (36)	0.47 (0.29-0.78)	0.003	0.60 (0.34-1.06)	0.08
No (n = 494)	46.8 (231)	1.0	---	1.0	---
**Time since diagnosis**					
< 12 months (n = 265)	52.8 (140)	1.0	---	---	---
12-24 months (n = 131)	37.4 (49)	0.53 (0.30-0.94)	0.03	0.61 (0.33-1.31)	0.12
2 months (n = 197)	35.0 (69)	0.48 (0.28-0.82)	0.01	0.74 (0.42-1.30)	0.31
**Knowledge: re-infection with new strain**					
Yes (n = 426)	36.8 (157)	1.0		1.0	
No/Does not know (n = 190)	57.9 (110)	2.35 (1.43-3.86)	0.001	1.27 (0.71-2.27)	0.41
**Disclosure to partner**					
Yes (n = 228)	31.1 (71)	1.0	---	---	---
No (n = 388)	50.5 (196)	2.26 (1.52-3.35)	< 0.001	2.38 (1.47-3.84)	< 0.001
**Transmission concerns**					
Yes (n = 394)	43.7 (172)	1.0	---		
No (n = 222)	42.8 (95)	0.97 (0.63-1.49)	0.87		
**Had STI in past 6 month**s					
Yes (n = 145)	42.8 (62)	0.97 (0.58-1.63)	0.91		
No (n = 471)	43.5 (205)	1.0	----		
**Perceived internalized stigma**					
Minimal/Low (n = 98)	17.4 (17)	1.0	---	---	---
Moderate (n = 431)	46.9 (202)	4.20 (2.22-7.95)	< 0.001	2.94 (1.50-5.75)	0.002
High (n = 87)	55.2 (48)	5.86 (2.60-13.21)	< 0.001	1.93 (0.74-5.03)	0.18
**Tired of using condoms**					
Agree (n = 253)	49.8 (126)	1.99 (1.26-3.14)	0.003	1.35 (0.80-2.25)	0.25
Disagree (n = 319)	33.2 (106)	1.0	---	---	---
Do not know(n = 44)	79.6 (35)	7.81 (3.31-18.43)	< 0.001	5.01 (1.78-14.07)	0.002
**Believe condom reduces pleasure**					
Agree (n = 369)	51.0 (188)	2.87 (1.81-4.54)	< 0.001	2.81 (1.60-4.91)	< 0.001
Disagree (n = 222)	26.6 (59)	1.0	---	---	---
Ambivalent (n = 25)	80.0 (20)	11.1 (4.03-30.28)	< 0.001	8.33 (2.38-29.09)	0.001
**Family planning**					
Using condom (n = 124)	16.1 (20)	1.0	---	---	---
Using other method (n = 117)	53.0 (62)	5.9 (2.87-11.96)	< 0.001	5.47 (2.57-11.66)	< 0.001
No family planning (n = 375	49.3 (185)	5.06 (2.72-9.42)	< 0.001	3.99 (2.06-7.75)	< 0.001
**Drink Alcohol Weekly**					
Yes (n = 251)	46.6 (117)	1.25 (0.79-1.97)	0.34		
No (n = 365)	41.1 (150)	1.0	---		

ART, antiretroviral therapy; CI, confidence interval; STI, sexually transmitted infection

In multivariate analysis, after controlling for multiple observations relating to different sexual partners reported by the same study participant, non-disclosure of HIV status to a partner (AOR 2.38, 95%CI: 1.47-3.84; p < 0.001), experiencing moderate levels of perceived stigma (AOR 2.94, 95%CI: 1.50-5.75; p = 0.002), believing condoms reduce sexual pleasure (AOR 2.81, 95%CI: 1.60-4.91; p < 0.001) or being unsure about condoms reducing pleasure (AOR 8.33, 95%CI: 2.38-29.09; p = 0.001), using a non-condom contraceptive method (AOR 5.47, 95%CI: 2.57-11.65; p < 0.001) or not using any contraception (AOR 3.99, 95%CI: 2.06-7.75; p < 0.001) were independently associated with US-6 months. Sex of the respondent, though not significantly associated with US-6 months in univariate analysis, was associated with US-6 months on multivariate analysis: female respondents were two times more likely to report US-6 months (AOR 2.10; 95%CI: 1.13-3.90; p = 0.018) compared to male respondents. University education and time since HIV diagnosis were not associated with US-6 months. Predictors for US-last sex were similar to those for US-6 months. (Data not shown)

### Sexually transmitted infections

Overall, 44% of participants reported ever having a STI other than HIV. Males were twice as likely to ever report a STI compared to females (55.9% vs. 41.0%; OR: 1.82, 95%CI: 1.27-2.61; p < 0.001). Of those who ever had a STI, half (49.5%) had a STI in the last 6 months. A higher proportion of female participants reported genital discharge (42.9% vs. 19.7%, OR: 3.06; 95%CI: 1.68-5.55; p < 0.001) and ulcers (38.2% vs. 25.5%; OR: 1.80; 95%CI: 1.04-3.11; p = 0.046) in the last six months than men. Of note, 46.5% of participants reporting a STI informed their regular partners of their infection, but only 13.9% of those with multiple partners informed other partners.

### Other sexual practices

Twenty nine percent of sexually active respondents reported sexual intercourse with a partner during menstruation (24/90 males and 94/318 females). Of those, 78% (18/24 males and 74/94 females) inconsistently or never used condoms for sex during menstrual periods. Eighteen percent of sexually active respondents (23/90 males and 50/318 females) reported ever having anal sex. Of those, 80.8% (14/23 males and 45/50 females) inconsistently or never used condoms during anal sex. (Data not shown)

## Discussion

This study, conducted in Mombasa among PLHIV not accessing HIV treatment, shows the population has high levels of unsafe sex. Almost sixty percent of the participants were sexually-active during the last 6 months. This is significantly higher than that reported in our previous study in Mombasa among PLHIV receiving ART (44%) and PLHIV receiving co-trimoxazole prophylaxis without ART (47%) [7], and in other studies among PLHIV accessing care services in Cote d' Ivoire (47%), Uganda (48%) and Cameroon (47%) [8,10,39]. Further, participants reported unprotected sex with more than half their sexual partners, significantly more with regular partners than non-regular partners. This is much higher than that reported among ART-naïve PLHIV in Uganda and South Africa [4,9,37] as well as among PLHIV on ART and those on co-trimoxazole prophylaxis without ART in Mombasa [2,7]. It is of concern that unprotected sex was reported with a third of HIV-negative partners and half of untested partners (people with unknown HIV status). This presents a serious HIV prevention challenge, particularly as 75 percent of the partners were untested and only 37 percent of the PLHIV had disclosed their HIV status to their partners. In a review article, Kalichman et al. (2000), have also reported high levels of unsafe sex with HIV-negative and unknown status partners [17]. Disclosure of HIV serostatus to partners and perceived stigma emerged as independent determinants of safe sex behaviors. It is important to note the intersection of the two determinants where PLHIV are reluctant to disclose for fear of rejection (perceived stigma) which may or may not happen [18,19]. Loubiere et al. (2009) and King et al. (2008) also link disclosure of HIV status with safe sex behavior in studies from Cameroon and Uganda [21,35]. Our study also highlights the role that the belief that condoms reduce pleasure and condom-use fatigue play in influencing safe sex. Conley and Collins (2005) found condom non-users to be more likely to believe that condom use interferes with pleasure; more commonly among males. Randolph et al. (2007) report similar results on condom use [40,41]. Prevention programs need to develop and implement strategies to change attitudes and beliefs about condoms. Further, more than half of the participants who did not want to have children were not using contraception, indicating high levels of unmet family planning need. Although it has been discussed extensively, effective integration of family planning counseling and services into HIV prevention programs has not been implemented and merits urgent action [42,43].

We documented other risky sexual practices such as unprotected sex during menstruation and unprotected anal sex. Sexual exposure to genital blood during menstruation is believed to facilitate transmission of HIV and other STIs [44,45]. We also report same sex behaviors among male participants: almost a quarter of all sexual partners reported by male participants were male. It is possible that a MSM peer could have recruited MSM participants. Mombasa has a fairly large population of male sex workers and unprotected anal sex is frequently reported in this population [46,47]. Anal intercourse is reported relatively less frequently by women. Fifteen percent of female participants in our study reported ever anal sex and the vast majority did not use condoms. Kalichman et al. (2009) report a 10% prevalence of heterosexual anal sex reported by women interviewed from community and clinic settings in South Africa [48]. The relatively low prevalence of anal intercourse among heterosexual individuals may be offset by its greater efficiency for transmitting HIV [49]. Health workers need to specifically discuss these forms of risky sexual behaviors during prevention counselling.

The study provides evidence that prevention programs can reach PLHIV who are not accessing HIV care services through community health workers or peer counsellors. About three-quarter of the participants were not accessing any HIV care and support services that they could benefit from; and more than half of them had been tested positive more than 12 months previously and were therefore, more likely to have forgotten any prevention messaging at the time of post-test counselling. This occurred despite the increased availability of HIV care services and ART in recent years. Further research is needed to examine why some PLHIV are not accessing HIV care services.

The study is not without limitations. We recruited participants using non-probability modified targeted snowball sampling. Although our sample is not a randomly recruited representative sample, this technique did allow us to reach PLHIV within the community who are otherwise not accessible. We believe we were able to recruit a sufficiently diverse and representative sample for this study. We did not use Respondent Driven Sampling, a technique used commonly for hidden hard-to-reach populations such as MSM and injecting drug users, as this sampling method relies heavily on the recruitment of peers through their social networks, and we felt this to be unsuitable for our population and setting. In Mombasa, the network of positive people is small and poorly organized and, our previous study showed that PLHIV were reluctant to reveal their status and were poorly networked, with high levels of internalized stigma. This has also been reported by other African studies [11,12,28]. Our study sample consisted of 76% female participants. There could be several reasons for this: women tend to stay at home and therefore may be more easily contacted by health workers, women may access care earlier than men and so are more likely to know their HIV status, and in general, women make up more than 60% of the HIV-positive population in sub-Saharan Africa [1]. Women constituted 64% and 66% of our sample in our two previous studies in Mombasa [2,7].

In our data analysis we did not control for clustering at the recruiter level, which could lead to increased variance in reported behaviors. We did not do so because we did not link data on individual recruiters to participants; we recorded only type of recruiters (CHW or PTC counsellor). However, the fact that we found no significant socio-demographic differences between PLHIV recruited by CHWs and those recruited by PCs, and that each health worker could bring in a limited number of participants into the study and health workers were able to reach different risk groups as there are no geographic areas in Mombasa with a concentration of particular high-risk populations, may have reduced the bias due to clustering at the recruiter level. The study relies on self-reported sexual risk and condom use behaviors which may be subject to social desirability and recall bias. For the partner level analysis, we limited the number of partners each participant could describe to a maximum of six in the reference period; this afforded us the ability to obtain more reliable recall and limit the influence of the outliers in the sample. Reviews of validity and reliability of HIV research have, however, found that sexual behavior data are fairly consistent and self-reported data on sexual acts are reasonably congruent, especially for infrequent acts and short recall periods [50,51]. However, recent studies using biomarkers to validate self-reported condom use suggest over reporting of condom use and recommend interpreting self-reported behaviors with caution [52]. Over reporting would further raise the level of risk found in this study. Finally, the study would have benefited if a control group of PLHIV on treatment had been included for a comparison of sexual behaviors.

In conclusion, a significantly large number of PLHIV in the community are not accessing ART or HIV care services in Mombasa and high risk sexual behaviors are widely prevalent in this population. HIV programs need to bring this population into the ambit of prevention and care services.

## Competing interests

The authors declare that they have no competing interests.

## Authors' contributions

AS was the PI on the study; she contributed to the design of the study, analysis of data and wrote the manuscript. SL participated in the design of the study and contributed to the manuscript. MP conducted the statistical data analysis. MC contributed substantially in reviewing the manuscript and guided data analysis. JO and NK conducted field research and assisted with contextual data and result interpretation. SG set up the data collection using hand held computers and helped with data interpretation. MT provided overall guidance for the research and manuscript preparation. All authors read and approved the final manuscript.
